# Doppler Ultrasound of the Umbilical Artery: Clinical Application

**DOI:** 10.1055/s-0042-1743097

**Published:** 2022-04-11

**Authors:** Ana Sá Rocha, Ana Rosa Araújo Andrade, Maria Lúcia Moleiro, Luís Guedes-Martins

**Affiliations:** 1 Instituto de Ciências Biomédicas Abel Salazar, Universidade do Porto, Porto, Portugal; 2 Departamento da Mulher e da Medicina Reprodutiva, Centro Materno Infantil do Norte, Centro Hospitalar Universitário do Porto EPE, Porto, Portugal; 3 Departamento da Mulher e da Medicina Reprodutiva, Centro Materno Infantil do Norte, Unidade de Investigação e Formação, Porto, Portugal; 4 Instituto de Investigação e Inovação em Saúde, Universidade do Porto, Porto, Portugal

**Keywords:** doppler, placenta, umbilical artery, fetal surveillance, placental insufficiency, doppler, placenta, artéria umbilical, vigilância fetal, insuficiência placentária

## Abstract

**Objective** To provide a survey of relevant literature on umbilical artery Doppler ultrasound use in clinical practice, technical considerations and limitations, and future perspectives.

**Methods** Literature searches were conducted in PubMed and Medline, restricted to articles written in English. Additionally, the references of all analyzed studies were searched to obtain necessary information.

**Results** The use of this technique as a routine surveillance method is only recommended for high-risk pregnancies with impaired placentation. Meta-analyses of randomized trials have established that obstetric management guided by umbilical artery Doppler findings can improve perinatal mortality and morbidity. The values of the indices of Umbilical artery Doppler decrease with advancing gestational age; however, a lack of consensus on reference ranges prevails.

**Conclusion** Important clinical decisions are based on the information obtained with umbilical artery Doppler ultrasound. Future efforts in research are imperative to overcome the current limitations of the technique.

## Resumo

**Objetivo** Compilar informação relevante proveniente da literatura atual sobre a ultrassonografia Doppler das artérias umbilicais (AUs) na prática clínica, considerações e limitações técnicas e perspectivas futuras.

**Métodos** A pesquisa bibliográfica foi realizada nos bancos de dados PubMed e Medline e restringiu-se a artigos escritos na língua inglesa. Recorreu-se também à bibliografia dos artigos selecionados, quando necessário, para obter informação relevante.

**Resultados** A utilização desta técnica como método de vigilância de rotina está apenas recomendada em gravidezes de alto risco com disfunção placentar. Metanálises de estudos randomizados mostraram que o seguimento obstétrico baseado nos achados do Doppler da artéria umbilical pode melhorar a mortalidade e a morbilidade perinatal. É consensual que os valores dos índices Doppler da AU decrescem com o avanço da idade gestacional. No entanto, há ainda muita incerteza quanto aos valores de referência.

**Conclusão** As informações obtidas através da AU Doppler US são a base para muitas decisões clínicas importantes. Trabalhos de investigação nesta área são essenciais para tentar colmatar atuais limitações da técnica.

## Introduction

The umbilical arteries (UAs) play a key role in the regulation of the fetoplacental circulation. In the UAs, nerve regulation is absent and its tonus depends uniquely on locally released or circulating vasoactive substances, as well as on ions, such as calcium (Ca ^2+^ ) and potassium (K ^+^ ). 1 2 3 4 5 6 7 They lead the deoxygenated blood from the fetus to the placenta during systole and diastole, and together with the umbilical vein, which conducts the blood on the opposite direction, the exchange of nutrients, respiratory gases, and metabolites between the mother and the fetus, is guaranteed. 8

To ensure normal intrauterine growth, there are some conditions that must be met: normal umbilical cord architecture and function; adequate placental perfusion; a healthy fetus and a favorable maternal condition; availability of nutrients and absence of pregnancy-related or non-related diseases. 1 8 9 Any abnormality in any of these prerequisites can potentially lead to intrauterine growth restriction (IUGR), with its inherent increased risk of perinatal mortality and morbidity in the short and long term. 1 9 10 11 12 13 14

The main cause of IUGR is placental insufficiency, 9 which is associated with an increased resistance to blood flow in the placental vasculature, restricting the blood supply to the fetus and inducing compensatory responses with hemodynamic changes. 9 15 16 The onset of IUGR can occur anytime during pregnancy, and strict fetal surveillance is required after the diagnosis to determine when staying in the womb represents a greater risk of adverse perinatal outcomes than being born. 10 17 18 19 20

Doppler ultrasound (US) of the UA provides useful information regarding the blood flow features within the arteries and is a well-established surveillance method in high-risk pregnancies due to impaired placentation. 11 20 21 22 In high-risk pregnancies, it is estimated that the use of Doppler US has allowed a decrease in the risk of perinatal death by ∼ 29%. 20

The physical principle behind the Doppler US technology is named after The Doppler Effect, which is defined as the variation in the frequencies transmitted to and received from US waves between two objects when at least one is moving. 23 24 In obstetrics, the constant object is the transducer, and the red blood cells of the uterofetoplacental circulation are the shifting reflectors that produce the returning signal echoes. 23

Spectral Doppler US is a speed-time spectral recording, presenting as flow velocity waveforms (FVWs). 25 It enables the quantification of the peak systolic velocity (PSV) and of the end-diastolic velocity (EDV) of blood flow within the UA, with which three indices can be obtained: the pulsatility index (PI), the resistance index (RI), and the systolic/diastolic ratio (S/D). 26 27 These indices are considered to be indirect measures of the resistance to blood flow of the placental vasculature. 1 11 28 29 30 Therefore, values not expected for the gestational age indicate placental dysfunction and fetal distress. 15 26 28 31

The UA Doppler US is widely used in fetal surveillance because it is a noninvasive, economical, simple, and reproducible method. 8 12 13 15 However useful, this technic has some limitations, including the potential to cause considerable anxiety in families and clinicians, further diagnostic testing, and early (possibly very preterm) birth. 11 Moreover, it has been found that many studies reporting reference ranges for UA Doppler are based in methodologies with much heterogeneity. 20 31

The aim of the present review is to provide a survey of the relevant literature on UA Doppler US in the clinical practice, its technical considerations and limitations, and to explore future perspectives.

## Methods

The present research aimed to include studies that focused on the applicability of UA Doppler US in pregnancy management. To compose the present review, thorough literature searches were conducted in the PubMed and Medline databases, restricted to articles written in the English language. The screening of articles was performed using the following terms from the Medical Subject Heading of the Index Medicus as keywords: *Doppler ultrasound* AND/OR *umbilical artery* . The list of obtained articles was revised and the ones dealing with placental evaluation, placental insufficiency, fetal/pregnancy surveillance, and IUGR were chosen for further revision. Articles found by cross-referencing that met the inclusion criteria were also included.

All identified studies were screened for these inclusion criteria: (1) published in English (2) with full-text available, (3) UA Doppler US application in pregnancy.

A selection of the articles was performed. First, articles were filtered by reviewing titles and abstracts using the same inclusion criteria. Second, the remaining articles were accessed based on the full text. Studies that did not meet all the inclusion criteria were excluded.

## Results

### Umbilical Artery Waveform Analysis

**Fig. 1:**
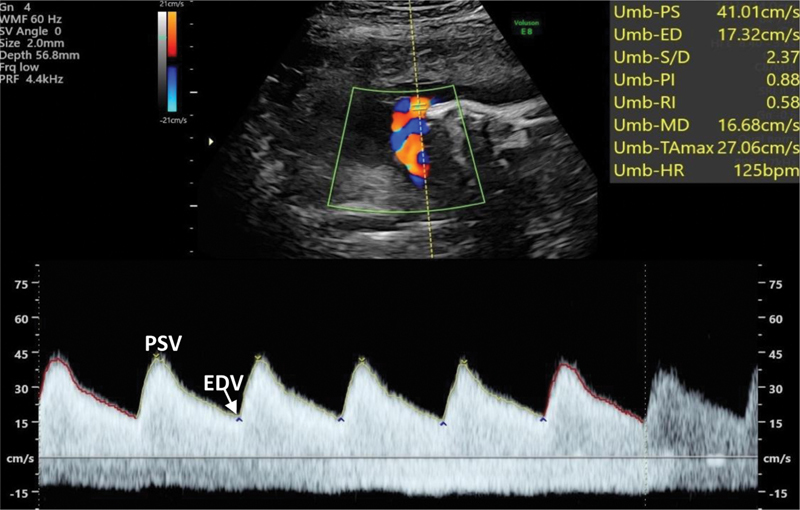
Normal umbilical artery flow velocity waveform tracings obtained during the 3 ^rd^ trimester. End diastolic velocities are present and are high; PSV - peak systolic velocity; EDV - end-diastolic velocity.

Concerning the UA, the standard Spectral Doppler US FVW pattern presents as a “sawtooth” pattern, revealing a unidirectional, continuous, and pulsatile flow toward the placenta ( Fig. 1 ). Its pattern can be distinguished from that of the umbilical vein since the UV FVW are continuous and nonpulsatile throughout the cardiac cycle. 32 33 In the “sawtooth” pattern of the UA, the highest point corresponds to the PSV, the lowest point corresponds to the EDV, and TAV stands for time-averaged velocity. These parameters enable the calculation of three indices: S/D Ratio: PSV/EDV; PI: (PSV - EDV)/ TAV; RI: (PSV - EDV) / PSV. 23 In the clinical practice, the PI is the most commonly used. 34

In low-risk pregnancies, the fetoplacental circulation presents itself with a placental high resistance to flow until the 20 ^th^ week; thereafter, it gradually decreases and becomes a low-resistance system. 8 This phenomenon occurs from the end of the 2 ^nd^ trimester due to the progressive placental villi maturation, greater width and wall compliance of the umbilical vessels along with greater fetal cardiac output and blood pressure. 35 36 Consequently, an acceleration in the EDV occurs and a proportional decrease in the three indices mentioned above is expected. 37 A deviation from the expected indices may signal an underlying placental dysfunction, and it indicates an increased risk of fetal demise, 31 38 39 40 regardless of the Doppler technique used. 35 41

**Fig. 2:**
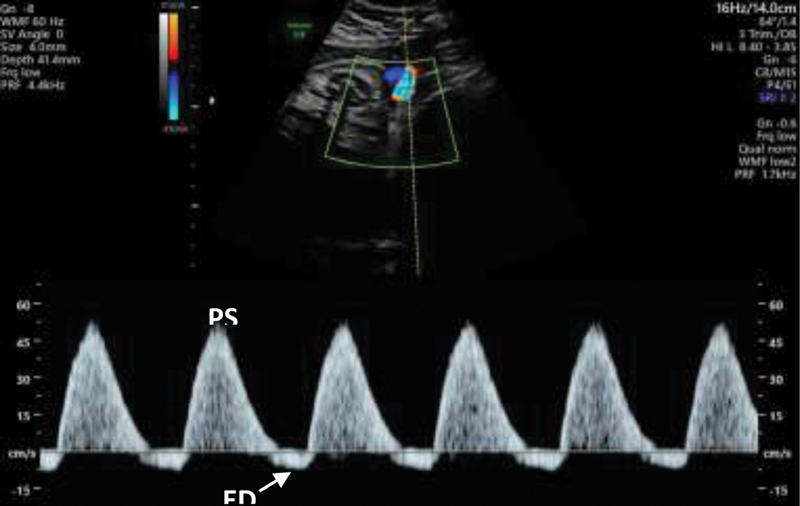
Abnormal umbilical artery flow velocity waveform tracings obtained during the 2 ^nd^ trimester. End diastolic velocities are absent, defining this pattern as AEDV. PSV - peak systolic velocity; EDV - end-diastolic velocity; AEDV - Absent end-diastolic velocity

**Fig. 3:**
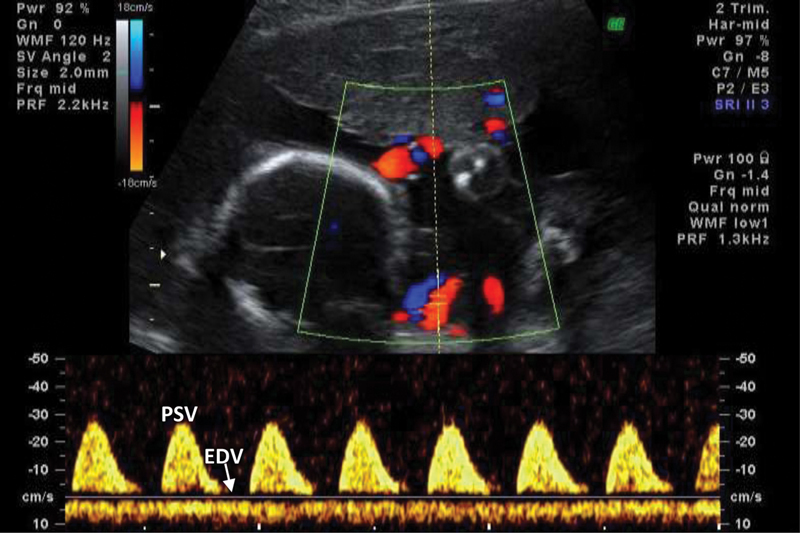
Abnormal umbilical artery flow velocity waveform tracings obtained in a 3 ^rd^ trimester pregnancy. End diastolic velocities are below the baseline, defining this pattern as REDV. PSV: peak systolic velocity; EDV: end-diastolic velocity; REDV: Reversed end-diastolic velocity

Pathological UA FVW has a progressive pattern of alterations, depending on the severity of the disorder: the EDV of the waveform becomes reduced (positive end-diastolic velocities [PEDV]), might disappear (absent end-diastolic velocities [AEDV]) ( Fig. 2 ), and can even reverse (reversed end-diastolic velocities [REDV]) ( Fig. 3 ), while PSV is not affected. 37 40 42 In these cases, the PI is more indicated for the interpretation of FVW findings 35 and it starts to increase only when 40% of the placental vascular tree remains functioning. 43

While an AEDV flow before the 15 ^th^ week is a normal physiological finding, 44 a REDV flow during the 1 ^st^ trimester is associated with chromosomal abnormalities, fetal cardiovascular defects, and significant mortality. 45 46 47 48 49 However, as stated by Bellver et al., 50 the latter “is not always an ominous sign.”

Once present, the AEDV can stabilize or gradually evolve to REDV. 51 In a small number of cases, an AEDV can ameliorate and normalize spontaneously around the 27 ^th^ week of gestation, although it is still unknown how to predict in which fetuses it will happen. 51 Antenatal administration of betamethasone to IUGR fetuses with absent or reversed end-diastolic velocity (AREDV) has also been correlated with the returning of the EDV and the stabilization of the resistance in the ductus venosus. By converting the AREDV to a normal flow, the outcome greatly improves, reverting the constant hypoxemia and acidosis to a better oxygenative status. 52 However, this positive effect of betamethasone is not seen in all cases, and the favorable response of the responding fetuses has not yet been understood. 52

Absent or reversed end-diastolic velocity is frequently associated with marginal placental-end cord insertion, 1 53 which can be accurately diagnosed by Color Doppler US during the 2 ^nd^ trimester. 12 Furthermore, in IUGR fetuses with AREDV, there is an increased expression of estrogen receptor-β within the fetoplacental endothelium, misbalancing the vascular tonus mediators and favoring vasoconstriction. 1 54 55 Being a vasodilator and smooth muscle relaxant, 56 the administration of intravenous or transdermal nitroglycerine causes a decrease in placental resistance to flow. This results in decreased PI, RI and S/D ratio in UA and Uterine artery (UtA) Doppler US, thus improving the outcomes. 56 57

When compared with PEDV, AREDV fetuses have a higher incidence of low birthweight, worse Apgar scores, and oligohydramnios; greater number of labor inductions and caesarean sections due to fetal distress; admissions to neonatal intensive care unit; fetal demise; perinatal mortality and morbidity, 58 59 60 61 62 as well as long-term neurological impairment. 14 63 64 65 The lower the gestational age and fetal weight at birth, the more severe are the neonatal complications. 58 Specifically, fetuses with trisomy 21 have higher prevalence of AREDV, along with the presence of maternal malperfusion, delayed villous maturation and fetal vascular malperfusion, shortened umbilical cord, congenital cardiac anomalies, which frequently result in growth restriction, and death *in utero* . 66

In IUGR fetuses, when in the presence of PEDV, an expectant attitude and close monitoring with weekly UA assessment is suggested, while in the presence of AREDV, after an acceptable gestational age is achieved, pregnancy termination seems to be the safest option to attain a better perinatal outcome. 37 58 Based on a recent meta-analysis, the 2021 International Federation of Gynecology and Obstetrics (FIGO) initiative on fetal growth suggested the application of UA Doppler findings as relative delivery criteria from 30 weeks onward for REDV and from 32 weeks onward for AEDV. 39 67

The analysis of FVW can alert obstetricians to other pathological entities in addition to placental disorders. A period of deceleration during a larger period of acceleration, or the opposite, is called *notching* . 68 A systolic notch in the UA FVW suggests the presence of an umbilical cord abnormality, such as an UA narrowing, an abnormal cord insertion, cord entanglement (in twin pregnancies) or a true knot. True knots, which are the major cause of notching, can impair the flow supply to the fetus and lead to adverse outcomes. The notching magnitude strongly correlates to how tight the knot is and it depends on the type of FVW being measured (envelope versus centerline), as well as on the location downstream of the constriction where the FVW is being measured. 68

Also worth of consideration are the results of a study conducted in 2006 by Struijk et al., 69 in which the magnitude-squared coherence function between the UtA and UA FVW was found to improve the early identification of preeclampsia during the mid-trimester. However, it has no applicability in the prediction of IUGR or of pregnancy-induced hypertension. 69

### Umbilical Artery Doppler Reference Ranges

**Fig. 4:**
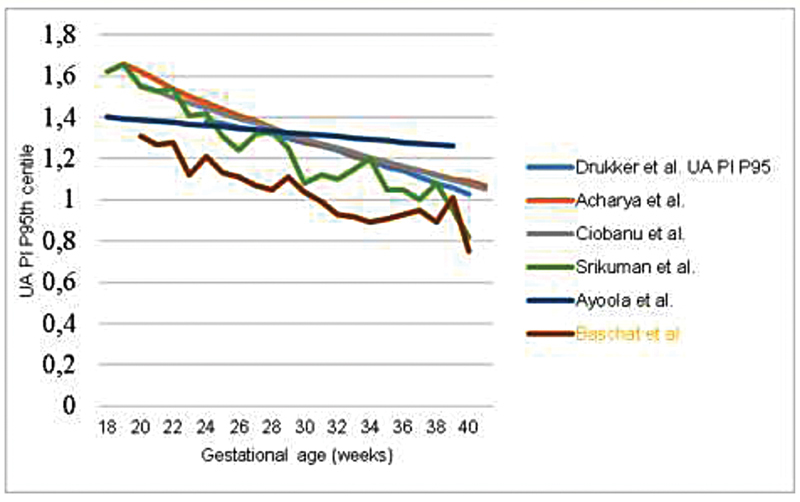
Comparison of the 95 ^th^ percentile of the umbilical artery pulsatility index in studies reporting reference ranges. UA: Umbilical artery; PI: Pulsatility index

There is a consensus that UA PI decreases linearly with advancing gestational age in uncomplicated singleton pregnancies. 15 31 35 70 71 72 73 74 75 ( Table 1 ) ( Fig. 4 ).

**Table 1:** Values of the 95 ^th^ centile for umbilical artery pulsatility index in studies reporting reference ranges

Gestational age (weeks)	Drukker et al. 72	Acharya et al. 73	Ciobanu et al. 71	Srikumar et al. 75	Ayoola et al. 74	Baschat et al. 76
18				1.62	1.402	
19		1.66		1.66	1.395	
20		1.62	1.553	1.55	1.388	1.31
21		1.58	1.526	1.53	1.381	1.27
22		1.54	1.499	1.54	1.375	1.28
23		1.5	1.472	1.41	1.368	1.12
24	1.38	1.47	1.446	1.42	1.361	1.21
25	1.37	1.44	1.42	1.31	1.354	1.13
26	1.35	1.41	1.395	1.24	1.348	1.11
27	1.34	1.38	1.371	1.32	1.341	1.07
28	1.32	1.35	1.346	1.33	1.334	1.05
29	1.3	1.32	1.322	1.25	1.327	1.11
30	1.28	1.29	1.299	1.08	1.321	1.04
31	1.26	1.27	1.275	1.12	1.314	0.99
32	1.24	1.25	1.252	1.1	1.307	0.93
33	1.21	1.22	1.229	1.15	1.3	0.92
34	1.19	1.2	1.207	1.2	1.294	0.89
35	1.16	1.18	1.184	1.05	1.287	0.91
36	1.14	1.16	1.162	1.05	1.28	0.93
37	1.11	1.14	1.14	1	1.273	0.95
38	1.08	1.12	1.118	1.08	1.267	0.89
39	1.06	1.1	1.097	0.95	1.26	1.01
40	1.03	1.09	1.075	0.82		0.75
41		1.07	1.053			

**Fig. 5:**
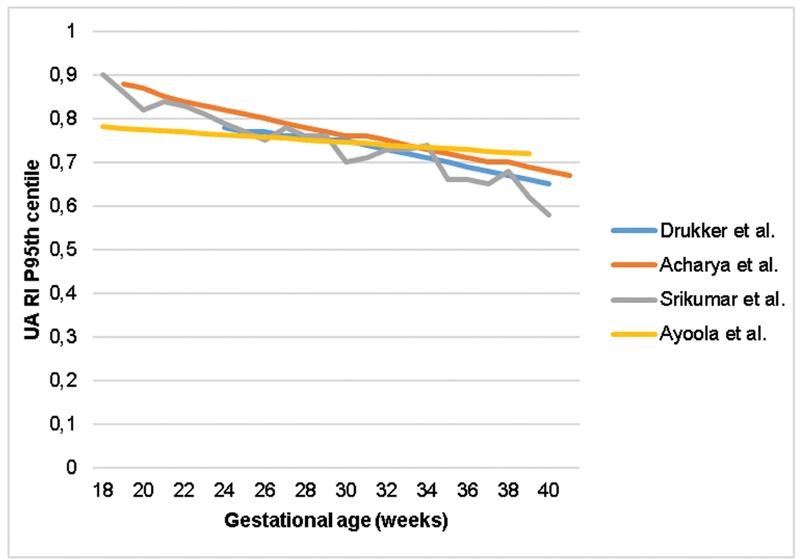
Comparison of the 95 ^th^ percentile of the umbilical artery resistance index in studies reporting reference ranges; UA: Umbilical artery; RI: Resistance index

However, the same percentile values were not obtained for each corresponding gestational age. 15 31 35 70 71 72 73 74 75 The same could be inferred about UA RI ( Table 2 ) ( Fig. 5 ). 72 73 74 75

**Table 2:** Values of the 95 ^th^ percentile for umbilical artery resistance index in studies reporting reference ranges

Gestational age (weeks)	Drukker et al. 72	Acharya et al. 73	Srikumar et al. 75	Ayoola et al. 74
18			0.9	0.781
19		0.88	0.86	0.778
20		0.87	0.82	0.775
21		0.85	0.84	0.772
22		0.84	0.83	0.769
23		0.83	0.81	0.766
24	0.78	0.82	0.79	0.763
25	0.77	0.81	0.77	0.76
26	0.77	0.8	0.75	0.758
27	0.76	0.79	0.78	0.755
28	0.76	0.78	0.76	0.752
29	0.75	0.77	0.76	0.749
30	0.75	0.76	0.7	0.746
31	0.74	0.76	0.71	0.743
32	0.73	0.75	0.73	0.74
33	0.72	0.74	0.73	0.737
34	0.71	0.73	0.74	0.734
35	0.7	0.72	0.66	0.732
36	0.69	0.71	0.66	0.729
37	0.68	0.7	0.65	0.726
38	0.67	0.7	0.68	0.723
39	0.66	0.69	0.62	0.72
40	0.65	0.68	0.58	
41		0.67		

Gathering values obtained in three different geographical areas, Drukker et al. 72 proposed universal charts for UA PI. They considered that uncomplicated pregnancies in excellent health, nutritional, and environmental conditions for fetal growth have similar fetoplacental function and, consequently, similar Doppler indices regardless of the country of origin and of the inherent characteristics of its population. 72 On the other hand, Ciobanu et al. 71 suggested that the *a priori* risk related to maternal characteristics and medical history should be taken into account as maternal age, body mass index, smoking, parity, and racial origin have significant impact on UA PI. Moreover, Widnes et al. 26 considered the influence of fetal gender and proposed gestational age-dependent gender reference ranges, as they found that female fetuses have a more pulsatile UA from the 20 ^th^ week to the 37 ^th^ week, and higher heart rates from the 26 ^th^ week.

In the case of fetuses with a single umbilical artery, Contro et al. 77 found the UA PI to be 20% lower than in those with a normal 3-vessel umbilical cord. This disparity remained constant between the 23 ^rd^ and 40 ^th^ gestational weeks. Thus, lower reference values in such cases may allow a more accurate interpretation of Doppler measurements. 77

Concerning twin pregnancies, Mulcahy et al. 78 described the UA PI and RI to be consistently higher, from early pregnancy, in both monochorionic (MC) and dichorionic (DC) twins in comparison with singletons. Also among twin pregnancies, MC twins tend to demonstrate slightly higher values of UA PI and RI compared with DC twins. 78 These findings are supported by Casati et al., 79 who proposed uncomplicated MC-specific Doppler charts, which include UA PI values. Since singleton Doppler reference ranges are not suitable for interpreting findings in twin pregnancies, further studies on both complicated and uncomplicated twin gestations and their perinatal and long-term outcomes are needed. 78 79

Maternal glucose loading 80 and fetal behavior state were found not to influence UA PI value measurements if adjusted to the fetal heart rate. 80 81 Although smoking during pregnancy is associated with an increased risk of adverse outcomes, 82 83 84 smoking habits seem not to influence fetal Doppler parameters. 85 A curious finding is that the left UA appears to have higher impedance to flow and as few as 2% of the pregnancies have both arteries with similar Doppler indices. 86

There is currently a wide variety of reference charts on UA Doppler indices, which could be explained, at least in part, by the heterogeneity in the methodological quality of the reports. Major methodological and statistical bias, found in some reports aiming to establish UA Doppler reference values, must be considered when examining this subject. 31 Even the studies with the highest methodological quality have significant discrepancy in cutoff values, which may signify important differences in clinical practice when using one cutoff value in preference to another. 31 When evaluating the potential impact of such variability on the clinical management of small for gestational age (SGA) fetuses, Ruiz-Martinez et al. 87 found the rate of labor inductions to vary from 2.1 to 33.7%, depending on which reference chart of the UA PI was used and considering the PI cutoff > 95 ^th^ percentile, as recommended in current clinical guidelines. 88 This example illustrates the magnitude of the impact that heterogeneous cutoff values have on decision-making in important clinical issues. 87 Another example is presented by Drukker et al., 72 who found the 95 ^th^ percentile values of UA PI to range between 1.28 and 1.48 at 32 weeks and between 1.03 and 1.40 at 39 weeks of pregnancy in different studies, illustrating a considerable uncertainty about what is a normal and expected cutoff value. 72

### Umbilical Artery Doppler as a Screening Test in Low-Risk Pregnancies

According to Alfirevic et al., 11 the methods traditionally used in low-risk pregnancies to assess fetal well-being (symphysis-fundal height measurement, fetal movements charts, and cardiotocography) have no proven ability to positively impact the low incidence and preventable adverse perinatal outcomes. Therefore, UA Doppler US was tested as a routine screening tool in low-risk pregnancies. In such pregnancies, UA Doppler US demonstrated low prognostic value concerning the risk of fetal demise, neonatal acidosis or decreased Apgar score. 89 Also, at term, an abnormal UA Doppler result in these cases can only have one consequence to improve the health of the newborn: intensified monitoring with possible elective delivery in the event of deteriorating fetal distress. 90 Considering its low predictable value and its cost of time, money and considerable anxiety of the parents, nowadays the routine screening of low-risk pregnancies with UA Doppler US is not recommended. 11 15 90 91

In contrast, according to Nkosi et al., 92 in developing countries and small centers with less financial resources, the routine use of Umbiflow (a continuous-wave Doppler machine) to screen low-risk pregnancies from the 28 ^th^ to the 32 ^nd^ week is beneficial. It allowed greater recognition of increased UA RI and AREDV patterns up to 5 to 10 times more than expected. 92 The identification of these fetuses at risk, among the until then considered low-risk pregnancies, led to an adequate and active management of those pregnancies and to an improvement in perinatal outcomes, avoiding several unexplained stillbirths. 92 93

Aiming to predict the perinatal outcome of low-risk pregnancies whose fetuses are suspected of IUGR, Gudmundsson et al. 94 proposed a new Doppler index: the placental pulsatility index. It combines the PI value of UA and UtA to evaluate the complete placental vascular impedance, and the authors suggest it has greater efficiency to predict adverse perinatal outcomes than UA and UtA alone. 94

### Umbilical Artery Doppler as a Screening Test in High-Risk Pregnancies

In contrast to low-risk pregnancies, the UA Doppler US is recommended as a routine surveillance method to assess fetal well-being in high-risk pregnancies. Especially in pregnancies complicated by placental dysfunction, as in IUGR or pre-eclampsia, UA Doppler US works as a predictive test for fetal compromise. 20 22 95 96 Its applicability in other high-risk groups such as diabetes mellitus, post-term, and uncomplicated dichorionic twin pregnancy is still uncertain. 20 97 98 99

The UA Doppler parameters are used to monitor fetal status and response to stress in pre-eclampsia and other hypertensive disorders related to pregnancy. However, it is the UtA PI that better predicts its future development 100 101 and anticipates adverse outcomes related to the condition. 102

Fetuses with estimated fetal weight (EFW) < 10 ^th^ centile are considered to be small for gestational age (SGA) and are at increased risk of fetal demise and poor perinatal outcomes when compared with non-SGA fetuses. 20 103 104 Some of these are constitutionally small healthy fetuses, whereas others are failing to reach their potential weight due to an underlying condition – IUGR fetuses. 11 20 105 Still, fetuses failing to reach their growth potential may or may not be SGA. 20 106

The criteria for diagnosing IUGR due to placental insufficiency include UA Doppler measurements. 107 There are 2 subtypes of IUGR, depending on whether the onset is before or after the 32 ^nd^ week, 107 both of which have distinguishable Doppler patterns and postnatal outcomes. 10 108 The early-onset IUGR (E-IUGR) is more frequently associated with early-onset pre-eclampsia 109 110 and a classical sequence of deterioration of Doppler indices is present. 111 112 113 114 First, the UA PI increases to abnormally high values and then the middle cerebral artery PI starts decreasing as the cardiovascular redistribution occurs. As the downstream impedance to flow keeps increasing, the EDV within the UA decreases and AREDV pattern settles down. These are followed by an abnormal ductus venosus FVW and fetal heart insufficiency. 111 112 113 114 The presence of an AREDV pattern or an EFW < 3 ^rd^ centile, before the 32 ^nd^ week, establishes the diagnosis of E-IUGR by itself. 107 In E-IUGR fetuses, the decision of labor induction based on fetal monitoring with non-stress test and ductus venosus Doppler seems to be associated with better results at 2 years of age. 17 38

The late-onset IUGR (L-IUGR) is more prevalent and has a lower mortality rate than E-IUGR 108 ; however, the undetected cases constitute the major cause of unexplained stillbirth. 11 103 115 In this subtype of IUGR, the UA Doppler indices remain unchanged or minimally elevated, not being reliable for diagnosis. 108 After the 32 ^nd^ week, the combination of biometrical parameters with Doppler measurements is more reliable than either one alone when differentiating the SGA at low-risk from those at high-risk for adverse outcomes. 108 These Doppler measurements must include the UA, the middle cerebral artery and the UtA as a multivessel screening in all pregnancies at high risk for placental dysfunction in the 3 ^rd^ trimester. 108 116 Finding both normal cerebroplacental ratio (CPR) and UtA Doppler indices, in fetuses presenting with an EFW > 3 ^rd^ centile, confirms the low-risk status and the managing protocol of constitutionally small fetuses is appropriate. 108 When Doppler indices suggest placental insufficiency (UA PI > 95 ^th^ centile or CPR < 5 ^th^ centile), an EFW < 10 ^th^ centile, or crossing > 2 quartiles on growth charts, has to be present to establish a high-risk status for late-SGA. However, an EFW < 3 ^rd^ centile alone, after the 32 ^nd^ week, establishes the diagnosis by itself. 107

Selective IUGR in DC twin pregnancies can also be monitored using UA Doppler US as it presents a flow progression pattern similar to that of IUGR in singleton pregnancies. In contrast, and due to the interdependent circulation, selective IUGR in MC twin pregnancies does not exhibit such pattern and the UA Doppler US is not a reliable tool to predict a possible deterioration of fetal status. 117 However, in MC pregnancies, a classification system based on the presence or absence of EDV in the UA in the affected twin guides its subsequent management. 117 118 Thus, twin pregnancies benefit from fetal well-being assessment with the UA Doppler US when there is a growth discordance, twin-to-twin transfusion syndrome, or IUGR. 119 120

In pregnancies complicated by gestational diabetes, 121 or with pre-existing diabetes mellitus without vascular disease, the non-stress test was found to be better than the UA Doppler US at predicting adverse perinatal outcomes. 98 121 Only those complicated with vasculopathy due to diabetes could benefit from periodic UA Doppler US monitoring. 98

## Discussion

The UA Doppler US has acquired an unquestionable importance as a fetal well-being surveillance method over the years and it is widely used in the clinical practice today.

In low-risk pregnancies, the placental impedance to flow is low and enables a continuous blood flow within the UA. 8 37 Placental insufficiency compromises this low-resistance system at the expense of the EDV. The higher the placental resistance, the lower the UA EDV, and the normal FVW “sawtooth” pattern progressively deteriorates into PEDV, AEDV, and ultimately into REDV patterns. These abnormal patterns are recognized as ominous and anticipatory signs of poor obstetric outcomes. 37 39 40 42 58 122 Likewise, the UA Doppler indices depend on EDV, and the PI, RI, and S/D ratio values are considered indirect measures of placental vasculature resistance to blood flow. 1 11 28 29 30

Concerning low-risk pregnancies, the routine use of UA Doppler US for fetal surveillance is not recommended. 11 90 91 Nonetheless, this assumption is based on studies conducted approximately 30 years ago. Therefore, it would be paramount to replicate these investigations with more accurate methodologies to determine whether there would be changes to the current knowledge or a corroboration of past conclusions.

In high-risk pregnancies, the UA Doppler US allows an accurate risk assessment for adverse outcomes and helps in the decision-making toward minimization of perinatal mortality and morbidity. 8 11 15 Current guidelines strongly recommend the routine use of this tool in high-risk pregnancies affected by placental insufficiency, such as those with IUGR and pregnancy-related hypertensive disorders. 20 22 95 96 However, during the 3 ^rd^ trimester, placental insufficiency develops under normal UA Doppler indices; 108 therefore, when suspected, other methods must be used to assess fetal well-being. 10 108 116 Regarding this issue, the TRUFFLE group is currently conducting a study (the TRUFFLE 2 study) aiming to address which monitoring methods and thresholds are ideal for determining the delivery of L-IUGR fetuses. 123 The role of UA Doppler US for fetal surveillance in high-risk pregnancies due to other precipitating factors requires further investigation. 20 31 97 98 99 124

Health improvements are not due to the application of the UA Doppler US itself but, rather, the result from the decision-making based on the information provided by this technology. Also, the success of Doppler measurements depends on the efficiency to spot abnormal and suspicious findings. Reference ranges are essential to establish which values of UA Doppler parameters must be considered normal and abnormal. Surprisingly, this is the point where less consensus exists. Although all studies agree that the values decrease with advancing gestational age, their proposed cutoff values differ significantly. 15 31 35 70 71 72 73 74 75 Studies on the methodological quality of reports proposing reference ranges have shown major methodological and statistical biases. 31 87 This may explain why so many different reference ranges have already been proposed. Another factor that may contribute to this variability is the wide range of variables that may influence UA Doppler indices. These can be fetal, maternal, or pregnancy-related variables, whose impact may be different when studied individually or in interaction. Given this and considering the potential impact of such variability on clinical decisions, the lack of consensus on reference ranges should incite scientific discussion. A universal chart was recently proposed aiming to standardize UA Doppler indices globally. 72 Although it sounds promising, future studies reporting its efficacy in different populations around the globe are paramount to state a conclusion.

## References

[1] Su E J Role of the fetoplacental endothelium in fetal growth restriction with abnormal umbilical artery Doppler velocimetry Am J Obstet Gynecol 2015 213(4, Suppl)S123 S130 10.1016/j.ajog.2015.06.038 26428491 PMC4592515

[2] Lorigo M Mariana M Feiteiro J Cairrao E How is the human umbilical artery regulated? J Obstet Gynaecol Res 2018 44 07 1193 1201 10.1111/jog.13667 29727040

[3] Salemme S Rebolledo A Speroni F Petruccelli S Milesi V L, P-/Q- and T-type Ca2+ channels in smooth muscle cells from human umbilical artery Cell Physiol Biochem 2007 20(1-4):55 64 10.1159/000104153 17595515

[4] Milesi V Raingo J Rebolledo A Grassi de Gende A O Potassium channels in human umbilical artery cells J Soc Gynecol Investig 2003 10 06 339 346 10.1016/s1071-5576(03)00117-5 12969776

[5] Reilly F D Russell P T Neurohistochemical evidence supporting an absence of adrenergic and cholinergic innervation in the human placenta and umbilical cord Anat Rec 1977 188 03 277 286 10.1002/ar.1091880302 900518

[6] Fox S B Khong T Y Lack of innervation of human umbilical cord. An immunohistological and histochemical study Placenta 1990 11 01 59 62 10.1016/s0143-4004(05)80443-6 2326237

[7] Poston L McCarthy A L Ritter J M Control of vascular resistance in the maternal and feto-placental arterial beds Pharmacol Ther 1995 65 02 215 239 10.1016/0163-7258(94)00064-a 7792316

[8] Krzyżanowski A Kwiatek M Gęca T Stupak A Kwaśniewska A Modern ultrasonography of the umbilical cord: prenatal diagnosis of umbilical cord abnormalities and assessement of fetal wellbeing Med Sci Monit 2019 25 3170 3180 10.12659/MSM.913762 31036798 PMC6505057

[9] Sankaran S Kyle P M Aetiology and pathogenesis of IUGR Best Pract Res Clin Obstet Gynaecol 2009 23 06 765 777 10.1016/j.bpobgyn.2009.05.003 19666240

[10] Mureșan D Rotar I C Stamatian F The usefulness of fetal Doppler evaluation in early versus late onset intrauterine growth restriction. Review of the literature Med Ultrason 2016 18 01 103 109 10.11152/mu.2013.2066.181.dop 26962562

[11] Alfirevic Z Stampalija T Medley N Fetal and umbilical Doppler ultrasound in normal pregnancy Cochrane Database Syst Rev 2015 04 CD001450 10.1002/14651858.CD001450.pub4 25874722 PMC6464774

[12] Sun J Wang L Li Y Clinical value of color doppler ultrasound in prenatal diagnosis of umbilical cord entry abnormity Pak J Med Sci 2016 32 06 1414 1418 10.12669/pjms.325.10518 28083036 PMC5216292

[13] Tanis J C Boelen M R Schmitz D M Casarella L van der Laan M E Bos A F Correlation between Doppler flow patterns in growth-restricted fetuses and neonatal circulation Ultrasound Obstet Gynecol 2016 48 02 210 216 10.1002/uog.15744 26358663

[14] Baschat A A Neurodevelopment after fetal growth restriction Fetal Diagn Ther 2014 36 02 136 142 10.1159/000353631 23886893

[15] Figueira C O Surita F G Dertkigil M S Pereira S L Bennini J R Jr. Morais S S Fetal Hemodynamic parameters in low risk pregnancies: Doppler velocimetry of uterine, umbilical, and middle cerebral artery ScientificWorldJournal 2016 2016 1.693704E6 10.1155/2016/1693704 PMC5124458 27957524

[16] Richardson B S Bocking A D Metabolic and circulatory adaptations to chronic hypoxia in the fetus Comp Biochem Physiol A Mol Integr Physiol 1998 119 03 717 723 10.1016/s1095-6433(98)01010-1 9683411

[17] TRUFFLE Investigators Frusca T Todros T Lees C Bilardo C M Outcome in early-onset fetal growth restriction is best combining computerized fetal heart rate analysis with ductus venosus Doppler: insights from the Trial of Umbilical and Fetal Flow in Europe Am J Obstet Gynecol 2018 218(2S, 2s):S783 S789 10.1016/j.ajog.2017.12.226 29422211

[18] Vollgraff Heidweiller-Schreurs C A De Boer M A Heymans M W Schoondmade L J Bossuyt P MM Mol B WJ Prognostic accuracy of cerebroplacental ratio and middle cerebral artery Doppler for adverse perinatal outcome: systematic review and meta-analysis Ultrasound Obstet Gynecol 2018 51 03 313 322 10.1002/uog.18809 28708272 PMC5873403

[19] Bakalis S Akolekar R Gallo D M Poon L C Nicolaides K H Umbilical and fetal middle cerebral artery Doppler at 30-34 weeks' gestation in the prediction of adverse perinatal outcome Ultrasound Obstet Gynecol 2015 45 04 409 420 10.1002/uog.14822 25684172

[20] Alfirevic Z Stampalija T Dowswell T Fetal and umbilical Doppler ultrasound in high-risk pregnancies Cochrane Database Syst Rev 2017 6 06 CD007529 10.1002/14651858.CD007529.pub4 28613398 PMC6481396

[21] American College of Obstetricians and Gynecologists' Committee on Practice Bulletins—Obstetrics and the Society forMaternal-FetalMedicin ACOG Practice Bulletin No. 204: fetal growth restriction Obstet Gynecol 2019 133 02 e97 e109 10.1097/AOG.0000000000003070 30681542

[22] Society for Maternal-Fetal Medicine Publications Committee Berkley E Chauhan S P Abuhamad A Doppler assessment of the fetus with intrauterine growth restriction Am J Obstet Gynecol 2012 206 04 300 308 10.1016/j.ajog.2012.01.022 22464066

[23] Oglat A A Matjafri M Z Suardi N Oqlat M A Abdelrahman M A Oqlat A A A review of medical Doppler ultrasonography of blood flow in general and especially in common carotid artery J Med Ultrasound 2018 26 01 3 13 10.4103/JMU.JMU_11_17 30065507 PMC6029191

[24] Moorthy R S Doppler ultrasound Med J Armed Forces India 2002 58 01 1 2 10.1016/S0377-1237(02)80001-6 27365648 PMC4923974

[25] Wood M M Romine L E Lee Y K Richman K M O'Boyle M Paz D A Spectral Doppler signature waveforms in ultrasonography: a review of normal and abnormal waveforms Ultrasound Q 2010 26 02 83 99 10.1097/RUQ.0b013e3181dcbf67 20498564

[26] Widnes C Flo K Wilsgaard T Kiserud T Acharya G Sex differences in umbilical artery Doppler indices: a longitudinal study Biol Sex Differ 2018 9 01 16 10.1186/s13293-018-0174-x 29669590 PMC5907403

[27] Nelson T R Pretorius D H The Doppler signal: where does it come from and what does it mean? AJR Am J Roentgenol 1988 151 03 439 447 10.2214/ajr.151.3.439 2970215

[28] Mone F McConnell B Thompson A Segurado R Hepper P Stewart M C Fetal umbilical artery Doppler pulsatility index and childhood neurocognitive outcome at 12 years BMJ Open 2016 6 06 e008916 10.1136/bmjopen-2015-008916 PMC4916642 27311899

[29] Trudinger B J Stevens D Connelly A Hales J R Alexander G Bradley L Umbilical artery flow velocity waveforms and placental resistance: the effects of embolization of the umbilical circulation Am J Obstet Gynecol 1987 157 06 1443 1448 10.1016/s0002-9378(87)80241-7 2962497

[30] Giles W B Trudinger B J Baird P J Fetal umbilical artery flow velocity waveforms and placental resistance: pathological correlation Br J Obstet Gynaecol 1985 92 01 31 38 10.1111/j.1471-0528.1985.tb01045.x 3966988

[31] Oros D Ruiz-Martinez S Staines-Urias E Conde-Aguiedo A Villar J Fabre E Reference ranges for Doppler indices of umbilical and fetal middle cerebral arteries and cerebroplacental ratio: systematic review Ultrasound Obstet Gynecol 2019 53 04 454 464 10.1002/uog.20102 30126005

[32] Hecher K Campbell S Characteristics of fetal venous blood flow under normal circumstances and during fetal disease Ultrasound Obstet Gynecol 1996 7 01 68 83 10.1046/j.1469-0705.1996.07010068.x 8932638

[33] Najafzadeh A Dickinson J E Umbilical venous blood flow and its measurement in the human fetus J Clin Ultrasound 2012 40 08 502 511 10.1002/jcu.21970 22855424

[34] Bhide A Acharya G Bilardo C M Brezinka C Cafici D Hernandez-Andrade E ISUOG practice guidelines: use of Doppler ultrasonography in obstetrics Ultrasound Obstet Gynecol 2013 41 02 233 239 10.1002/uog.12371 23371348

[35] Bahlmann F Fittschen M Reinhard I Wellek S Puhl A Blood flow velocity waveforms of the umbilical artery in a normal population: reference values from 18 weeks to 42 weeks of gestation Ultraschall Med 2012 33 07 E80 E87 10.1055/s-0031-1299294 22331834

[36] Trudinger B J Giles W B Cook C M Bombardieri J Collins L Fetal umbilical artery flow velocity waveforms and placental resistance: clinical significance Br J Obstet Gynaecol 1985 92 01 23 30 10.1111/j.1471-0528.1985.tb01044.x 4038455

[37] Todros T Piccoli E Rolfo A Cardaropoli S Guiot C Gaglioti P Review: Feto-placental vascularization: a multifaceted approach Placenta 2011 32 02 S165 S169 10.1016/j.placenta.2010.12.020 21232791

[38] TRUFFLE Group Lees C Marlow N Arabin B Bilardo C M Brezinka C Derks J B Perinatal morbidity and mortality in early-onset fetal growth restriction: cohort outcomes of the trial of randomized umbilical and fetal flow in Europe (TRUFFLE) Ultrasound Obstet Gynecol 2013 42 04 400 408 10.1002/uog.13190 24078432

[39] Caradeux J Martinez-Portilla R J Basuki T R Kiserud T Figueras F Risk of fetal death in growth-restricted fetuses with umbilical and/or ductus venosus absent or reversed end-diastolic velocities before 34 weeks of gestation: a systematic review and meta-analysis Am J Obstet Gynecol 2018 218(2S):S774 82.e21, 782.e21 10.1016/j.ajog.2017.11.566 29233550

[40] Kalache K D Dückelmann A M Doppler in obstetrics: beyond the umbilical artery Clin Obstet Gynecol 2012 55 01 288 295 10.1097/GRF.0b013e3182488156 22343245

[41] Giles W B Lingman G Marsál K Trudinger B J Fetal volume blood flow and umbilical artery flow velocity waveform analysis: a comparison Br J Obstet Gynaecol 1986 93 05 461 465 3707876

[42] Marsál K Rational use of Doppler ultrasound in perinatal medicine J Perinat Med 1994 22 06 463 474 7674100

[43] Thompson R S Trudinger B J Doppler waveform pulsatility index and resistance, pressure and flow in the umbilical placental circulation: an investigation using a mathematical model Ultrasound Med Biol 1990 16 05 449 458 10.1016/0301-5629(90)90167-b 2238251

[44] Fisk N M MacLachlan N Ellis C Tannirandorn Y Tonge H M Rodeck C H Absent end-diastolic flow in first trimester umbilical artery Lancet 1988 2(8622):1256 1257 10.1016/s0140-6736(88)90854-9 2903991

[45] Borrell A Martinez J M Farre M T Azulay M Cararach V Fortuny A Reversed end-diastolic flow in first-trimester umbilical artery: an ominous new sign for fetal outcome Am J Obstet Gynecol 2001 185 01 204 207 10.1067/mob.2001.114872 11483929

[46] Borrell A Costa D Martinez J M Farré M T Palacio M Mortera C Fortuny A Reversed end-diastolic umbilical flow in a first-trimester fetus with congenital heart disease Prenat Diagn 1998 18 10 1001 1005 10.1002/(sici)1097-0223(1998100)18:10<1001:aid-pd395>3.0.co;2-v 9826890

[47] Martinez Crespo J M Comas C Borrell A Puerto B Antolin E Ojuel J Reversed end-diastolic umbilical artery velocity in two cases of trisomy 18 at 10 weeks' gestation Ultrasound Obstet Gynecol 1996 7 06 447 449 10.1046/j.1469-0705.1996.07060447.x 8807764

[48] Murta C G Moron A F Avila M A Reversed diastolic umbilical artery flow in the first trimester associated with chromosomal fetal abnormalities or cardiac defects Obstet Gynecol 2000 95(6 Pt 2):1011 1013 10.1016/s0029-7844(99)00603-1 10808007

[49] Comas C Carrera M Devesa R Muñoz A Torrents M Ribas I Early detection of reversed diastolic umbilical flow: should we offer karyotyping? Ultrasound Obstet Gynecol 1997 10 06 400 402 10.1046/j.1469-0705.1997.10060400.x 9476324

[50] Bellver J Lara C Rossal L P Remohí J Pellicer A Serra V First-trimester reversed end-diastolic flow in the umbilical artery is not always an ominous sign Ultrasound Obstet Gynecol 2003 22 06 652 655 10.1002/uog.922 14689543

[51] Simonazzi G Curti A Cattani L Rizzo N Pilu G Outcome of severe placental insufficiency with abnormal umbilical artery Doppler prior to fetal viability BJOG 2013 120 06 754 757 10.1111/1471-0528.12133 23320863

[52] Piazze J Dillon K C Cerekja A Betamethasone effects on umbilical arteries and ductus venosus Doppler velocity waveforms in growth-restricted fetuses J Matern Fetal Neonatal Med 2012 25 07 1179 1182 10.3109/14767058.2011.624216 21923610

[53] Nordenvall M Ullberg U Laurin J Lingman G Sandstedt B Ulmsten U Placental morphology in relation to umbilical artery blood velocity waveforms Eur J Obstet Gynecol Reprod Biol 1991 40 03 179 190 10.1016/0028-2243(91)90115-2 1879593

[54] Su E J Ernst L Abdallah N Chatterton R Xin H Monsivais D Estrogen receptor-β and fetoplacental endothelial prostanoid biosynthesis: a link to clinically demonstrated fetal growth restriction J Clin Endocrinol Metab 2011 96 10 E1558 E1567 10.1210/jc.2011-1084 21832119 PMC3200254

[55] Reierstad Su E J Lin Z H Zeine R Yin P Innes J E Estrogen receptor-beta mediates cyclooxygenase-2 expression and vascular prostanoid levels in human placental villous endothelial cells Am J Obstet Gynecol 2009 200 04 4270 4.27E10 10.1016/j.ajog.2009.01.025 19318151

[56] Gupta S Chauhan M Sen J Nanda S Effect of transdermal nitroglycerine on Doppler velocity waveforms of the uterine, umbilical and fetal middle cerebral arteries in patients with chronic placental insufficiency: a prospective RCT J Clin Diagn Res 2017 11 07 QC13 QC17 10.7860/JCDR/2017/21438.10282 PMC5583822 28892981

[57] Mishra M Sawhney R Kumar A Bapna K R Kohli V Wasir H Cardiac surgery during pregnancy: continuous fetal monitoring using umbilical artery Doppler flow velocity indices Ann Card Anaesth 2014 17 01 46 51 10.4103/0971-9784.124141 24401303

[58] Yildirim G Turhan E Aslan H Gungorduk K Guven H Idem O Perinatal and neonatal outcomes of growth restricted fetuses with positive end diastolic and absent or reversed umbilical artery doppler waveforms Saudi Med J 2008 29 03 403 408 18327368

[59] Karsdorp V H van Vugt J M van Geijn H P Kostene P J Arduini D Montenegro N Clinical significance of absent or reversed end diastolic velocity waveforms in umbilical artery Lancet 1994 344(8938):1664 1668 10.1016/s0140-6736(94)90457-x 7996959

[60] Al Hamayel N A Baghlaf H Blakemore K Crino J P Burd I Significance of abnormal umbilical artery Doppler studies in normally grown fetuses Matern Health Neonatol Perinatol 2020 6 1 10.1186/s40748-020-0115-7 32110420 PMC7033920

[61] Tolu L B Ararso R Abdulkadir A Feyissa G T Worku Y Perinatal outcome of growth restricted fetuses with abnormal umbilical artery Doppler waveforms compared to growth restricted fetuses with normal umbilical artery Doppler waveforms at a tertiary referral hospital in urban Ethiopia PLoS One 2020 15 06 e0234810 10.1371/journal.pone.0234810 32555633 PMC7302535

[62] Byun Y J Kim H S Yang J I Kim J H Kim H Y Chang S J Umbilical artery Doppler study as a predictive marker of perinatal outcome in preterm small for gestational age infants Yonsei Med J 2009 50 01 39 44 10.3349/ymj.2009.50.1.39 19259346 PMC2649859

[63] Valcamonico A Danti L Frusca T Soregaroli M Zucca S Abrami F Absent end-diastolic velocity in umbilical artery: risk of neonatal morbidity and brain damage Am J Obstet Gynecol 1994 170 03 796 801 10.1016/s0002-9378(94)70285-3 8141204

[64] Valcamonico A Accorsi P Battaglia S Soregaroli M Beretta D Frusca T Absent or reverse end-diastolic flow in the umbilical artery: intellectual development at school age Eur J Obstet Gynecol Reprod Biol 2004 114 01 23 28 10.1016/j.ejogrb.2003.09.033 15099866

[65] Brütsch S Burkhardt T Kurmanavicius J Bassler D Zimmermann R Natalucci G Neurodevelopmental outcome in very low birthweight infants with pathological umbilical artery flow Arch Dis Child Fetal Neonatal Ed 2016 101 03 F212 F216 10.1136/archdischild-2014-307820 26304460

[66] Corry E Mone F Segurado R Downey P McParland P McAuliffe F M Placental disease and abnormal umbilical artery Doppler waveforms in trisomy 21 pregnancy: A case-control study Placenta 2016 47 24 28 10.1016/j.placenta.2016.09.001 27780536

[67] Melamed N Baschat A Yinon Y Athanasiadis A Mecacci F Figueras F FIGO (international Federation of Gynecology and obstetrics) initiative on fetal growth: best practice advice for screening, diagnosis, and management of fetal growth restriction Int J Gynaecol Obstet 2021 152 01 3 57 10.1002/ijgo.13522 33740264 PMC8252743

[68] Tejada-Martínez A E Borberg C J Venugopal R Carballo C Moreno W A Quintero R A Computational fluid dynamic analysis of flow velocity waveform notching in umbilical arteries Am J Physiol Regul Integr Comp Physiol 2011 300 01 R76 R84 10.1152/ajpregu.00864.2009 20926769

[69] Struijk P C Fernando K L Mathews V J Steegers E AP Wladimiroff J W Clark E B Application of the magnitude-squared coherence function between uterine and umbilical flow velocity waveforms for predicting placental dysfunction: a preliminary study Ultrasound Med Biol 2007 33 07 1057 1063 10.1016/j.ultrasmedbio.2007.01.012 17448590

[70] Dias T Abeykoon S Mendis P Gunawardena C Pragasan G Padeniya T Fetal Doppler reference values in women with a normal body mass index Ceylon Med J 2019 64 02 59 65 10.4038/cmj.v64i2.8888 31455068

[71] Ciobanu A Wright A Syngelaki A Wright D Akolekar R Nicolaides K H Fetal Medicine Foundation reference ranges for umbilical artery and middle cerebral artery pulsatility index and cerebroplacental ratio Ultrasound Obstet Gynecol 2019 53 04 465 472 10.1002/uog.20157 30353583

[72] Drukker L Staines-Urias E Villar J Barros F C Carvalho M Munim S International gestational age-specific centiles for umbilical artery Doppler indices: a longitudinal prospective cohort study of the INTERGROWTH-21 ^st^ Project Am J Obstet Gynecol 2020 222 06 6020 6.02E17 10.1016/j.ajog.2020.01.012 PMC7287403 31954701

[73] Acharya G Wilsgaard T Berntsen G K Maltau J M Kiserud T Reference ranges for serial measurements of umbilical artery Doppler indices in the second half of pregnancy Am J Obstet Gynecol 2005 192 03 937 944 10.1016/j.ajog.2004.09.019 15746695

[74] Ayoola O O Bulus P Loto O M Idowu B M Normogram of umbilical artery Doppler indices in singleton pregnancies in south-western Nigerian women J Obstet Gynaecol Res 2016 42 12 1694 1698 10.1111/jog.13114 27762476

[75] Srikumar S Debnath J Ravikumar R Bandhu H C Maurya V K Doppler indices of the umbilical and fetal middle cerebral artery at 18-40 weeks of normal gestation: A pilot study Med J Armed Forces India 2017 73 03 232 241 10.1016/j.mjafi.2016.12.008 28790780 PMC5533518

[76] Baschat A A Gembruch U The cerebroplacental Doppler ratio revisited Ultrasound Obstet Gynecol 2003 21 02 124 127 10.1002/uog.20 12601831

[77] Contro E Cataneo I Morano D Farina A Reference charts for umbilical Doppler pulsatility index in fetuses with isolated two-vessel cord Arch Gynecol Obstet 2019 299 04 947 951 10.1007/s00404-019-05086-z 30730012

[78] Mulcahy C McAuliffe F M Breathnach F Geary M Daly S Higgins J Umbilical and fetal middle cerebral artery Doppler reference ranges in a twin population followed longitudinally from 24 to 38 weeks' gestation Ultrasound Obstet Gynecol 2014 44 04 461 467 10.1002/uog.13302 24407772

[79] Casati D Pellegrino M Cortinovis I Spada E Lanna M Faiola S Longitudinal Doppler references for monochorionic twins and comparison with singletons PLoS One 2019 14 12 e0226090 10.1371/journal.pone.0226090 31809530 PMC6897428

[80] Haugen G Bollerslev J Henriksen T Human umbilical and fetal cerebral blood flow velocity waveforms following maternal glucose loading: a cross-sectional observational study Acta Obstet Gynecol Scand 2016 95 06 683 689 10.1111/aogs.12913 27099205

[81] Mulders L G Muijsers G J Jongsma H W Nijhuis J G Hein P R The umbilical artery blood flow velocity waveform in relation to fetal breathing movements, fetal heart rate and fetal behavioural states in normal pregnancy at 37 to 39 weeks Early Hum Dev 1986 14(3-4):283 293 10.1016/0378-3782(86)90191-x 3803274

[82] Soneji S Beltrán-Sánchez H Association of maternal cigarette smoking and smoking cessation with preterm birth JAMA Netw Open 2019 2 04 e192514 10.1001/jamanetworkopen.2019.2514 31002320 PMC6481448

[83] Tikkanen M Nuutila M Hiilesmaa V Paavonen J Ylikorkala O Prepregnancy risk factors for placental abruption Acta Obstet Gynecol Scand 2006 85 01 40 44 10.1080/00016340500324241 16521678

[84] Reeves S Bernstein I Effects of maternal tobacco-smoke exposure on fetal growth and neonatal size Expert Rev Obstet Gynecol 2008 3 06 719 730 10.1586/17474108.3.6.719 19881889 PMC2770192

[85] Stalzer A Seybold D Hossino D Broce M Calhoun B Doppler screening and predictors of adverse outcomes in high risk pregnancies affected by tobacco Reprod Toxicol 2017 67 10 14 10.1016/j.reprotox.2016.11.006 27836536

[86] Janeczek S Karlman R MacMillan W Left versus right intra-abdominal umbilical arteries: comparison of their Doppler waveforms J Ultrasound Med 2012 31 05 679 683 10.7863/jum.2012.31.5.67 22535714

[87] Ruiz-Martinez S Papageorghiou A T Staines-Urias E Villar J Gonzalez de Agüero R Oros D Clinical impact of Doppler reference charts to manage fetal growth restriction: need for standardization Ultrasound Obstet Gynecol 2020 56 02 166 172 10.1002/uog.20380 31237023

[88] ACOG Practice bulletin no. 134: fetal growth restriction Obstet Gynecol 2013 121 05 1122 1133 10.1097/01.AOG.0000429658.85846.f9 23635765

[89] Bolz N Kalache K D Proquitte H Slowinski T Hartung J P Henrich W Value of Doppler sonography near term: can umbilical and uterine artery indices in low-risk pregnancies predict perinatal outcome? J Perinat Med 2013 41 02 165 170 10.1515/jpm-2012-0042 23096449

[90] Doppler French Study Group A randomised controlled trial of Doppler ultrasound velocimetry of the umbilical artery in low risk pregnancies Br J Obstet Gynaecol 1997 104 04 419 424 10.1111/j.1471-0528.1997.tb11492.x 9141577

[91] Goffinet F Paris-Llado J Nisand I Bréart G Umbilical artery Doppler velocimetry in unselected and low risk pregnancies: a review of randomised controlled trials Br J Obstet Gynaecol 1997 104 04 425 430 10.1111/j.1471-0528.1997.tb11493.x 9141578

[92] Nkosi S Makin J Hlongwane T Pattinson R C Screening and managing a low-risk pregnant population using continuous-wave Doppler ultrasound in a low-income population: A cohort analytical study S Afr Med J 2019 109 05 347 352 10.7196/SAMJ.2019.v109i5.13611 31131803

[93] Hugo E J Odendaal H J Grove D Evaluation of the use of umbilical artery Doppler flow studies and outcome of pregnancies at a secondary hospital J Matern Fetal Neonatal Med 2007 20 03 233 239 10.1080/14767050601134926 17437225

[94] Gudmundsson S Flo K Ghosh G Wilsgaard T Acharya G Placental pulsatility index: a new, more sensitive parameter for predicting adverse outcome in pregnancies suspected of fetal growth restriction Acta Obstet Gynecol Scand 2017 96 02 216 222 10.1111/aogs.13060 27858967

[95] FitzGerald D E Drumm J E Non-invasive measurement of human fetal circulation using ultrasound: a new method BMJ 1977 2(6100):1450 1451 10.1136/bmj.2.6100.1450 589262 PMC1632644

[96] Morris R K Malin G Robson S C Kleijnen J Zamora J Khan K S Fetal umbilical artery Doppler to predict compromise of fetal/neonatal wellbeing in a high-risk population: systematic review and bivariate meta-analysis Ultrasound Obstet Gynecol 2011 37 02 135 142 10.1002/uog.7767 20922778

[97] French College of Gynaecologists and Obstetricians Vayssière C Benoist G Blondel B Deruelle P Favre R Gallot D Twin pregnancies: guidelines for clinical practice from the French College of Gynaecologists and Obstetricians (CNGOF) Eur J Obstet Gynecol Reprod Biol 2011 156 01 12 17 10.1016/j.ejogrb.2010.12.045 21277672

[98] Graves C R Antepartum fetal surveillance and timing of delivery in the pregnancy complicated by diabetes mellitus Clin Obstet Gynecol 2007 50 04 1007 1013 10.1097/GRF.0b013e31815a63cc 17982344

[99] de Rochambeau B Jabbour N Mellier G [Umbilical Doppler velocimetry in prolonged pregnancies] Rev Fr Gynécol Obstet 1992 87 05 289 294French. 1626175

[100] Adekanmi A J Roberts A Akinmoladun J A Adeyinka A O Uterine and umbilical artery doppler in women with pre-eclampsia and their pregnancy outcomes Niger Postgrad Med J 2019 26 02 106 112 10.4103/npmj.npmj_161_18 31187750

[101] Velauthar L Plana M N Kalidindi M Zammora J Thilaganathan B Illanes S E First-trimester uterine artery Doppler and adverse pregnancy outcome: a meta-analysis involving 55,974 women Ultrasound Obstet Gynecol 2014 43 05 500 507 10.1002/uog.13275 24339044

[102] Shen G Huang Y Jiang L Gu J Wang Y Hu B Ultrasound prediction of abnormal infant development in hypertensive pregnant women in the second and third trimester Sci Rep 2017 7 40429 10.1038/srep40429 28091544 PMC5238445

[103] Gardosi J Madurasinghe V Williams M Malik A Francis A Maternal and fetal risk factors for stillbirth: population based study BMJ 2013 346 f108 10.1136/bmj.f108 23349424 PMC3554866

[104] Pilliod R A Cheng Y W Snowden J M Doss A E Caughey A B The risk of intrauterine fetal death in the small-for-gestational-age fetus Am J Obstet Gynecol 2012 207 04 3180 3.18E8 10.1016/j.ajog.2012.06.039 PMC3724359 23021697

[105] Figueras F Gardosi J Intrauterine growth restriction: new concepts in antenatal surveillance, diagnosis, and management Am J Obstet Gynecol 2011 204 04 288 300 10.1016/j.ajog.2010.08.055 21215383

[106] Bardien N Whitehead C L Tong S Ugoni A McDonald S Walker S P Placental insufficiency in fetuses that slow in growth but are born appropriate for gestational age: a prospective longitudinal study PLoS One 2016 11 01 e0142788 10.1371/journal.pone.0142788 26730589 PMC4701438

[107] Gordijn S J Beune I M Thilaganathan B Papageorghiou A Baschat A A Baker N Consensus definition of fetal growth restriction: a Delphi procedure Ultrasound Obstet Gynecol 2016 48 03 333 339 10.1002/uog.15884 26909664

[108] Figueras F Caradeux J Crispi F Eixarch E Peguero A Gratacos E Diagnosis and surveillance of late-onset fetal growth restriction Am J Obstet Gynecol 2018 218(2S, 2s):S790, 802.e1 10.1016/j.ajog.2017.12.003 29422212

[109] Mifsud W Sebire N J Placental pathology in early-onset and late-onset fetal growth restriction Fetal Diagn Ther 2014 36 02 117 128 10.1159/000359969 24577279

[110] Yinon Y Kingdom J C Odutayo A Moineddin R Drewlo S Lai V Vascular dysfunction in women with a history of preeclampsia and intrauterine growth restriction: insights into future vascular risk Circulation 2010 122 18 1846 1853 10.1161/CIRCULATIONAHA.110.948455 20956209

[111] Turan O M Turan S Gungor S Berg C Moyano D Gembruch U Progression of Doppler abnormalities in intrauterine growth restriction Ultrasound Obstet Gynecol 2008 32 02 160 167 10.1002/uog.5386 18634130

[112] Baschat A A Gembruch U Harman C R The sequence of changes in Doppler and biophysical parameters as severe fetal growth restriction worsens Ultrasound Obstet Gynecol 2001 18 06 571 577 10.1046/j.0960-7692.2001.00591.x 11844191

[113] Hecher K Bilardo C M Stigter R H Ville Y Hackelöer B J Kok H J Monitoring of fetuses with intrauterine growth restriction: a longitudinal study Ultrasound Obstet Gynecol 2001 18 06 564 570 10.1046/j.0960-7692.2001.00590.x 11844190

[114] Ferrazzi E Bozzo M Rigano S Bellotti M Morabito A Pardi G Temporal sequence of abnormal Doppler changes in the peripheral and central circulatory systems of the severely growth-restricted fetus Ultrasound Obstet Gynecol 2002 19 02 140 146 10.1046/j.0960-7692.2002.00627.x 11876805

[115] Gardosi J Kady S M McGeown P Francis A Tonks A Classification of stillbirth by relevant condition at death (ReCoDe): population based cohort study BMJ 2005 331(7525):1113 1117 10.1136/bmj.38629.587639.7C 16236774 PMC1283273

[116] Geerts L Van der Merwe E Theron A Rademan K Placental insufficiency among high-risk pregnancies with a normal umbilical artery resistance index after 32weeks Int J Gynaecol Obstet 2016 135 01 38 42 10.1016/j.ijgo.2016.03.038 27515046

[117] Townsend R Khalil A Twin pregnancy complicated by selective growth restriction Curr Opin Obstet Gynecol 2016 28 06 485 491 10.1097/GCO.0000000000000326 27755130

[118] Gratacós E Lewi L Muñoz B Acosta-Rojas R Hernandez-Andrade E Martinez J M A classification system for selective intrauterine growth restriction in monochorionic pregnancies according to umbilical artery Doppler flow in the smaller twin Ultrasound Obstet Gynecol 2007 30 01 28 34 10.1002/uog.4046 17542039

[119] Maulik D Mundy D Heitmann E Maulik D Evidence-based approach to umbilical artery Doppler fetal surveillance in high-risk pregnancies: an update Clin Obstet Gynecol 2010 53 04 869 878 10.1097/GRF.0b013e3181fbb5f5 21048454

[120] DIAGNOSTIC IMAGING COMMITTEE SPECIAL CONTRIBUTOR GENETICS COMMITTEE MATERNAL FETAL MEDICINE COMMITTEE Morin L Lim K Ultrasound in twin pregnancies J Obstet Gynaecol Can 2011 33 06 643 656 10.1016/S1701-2163(16)34916-7 21846456

[121] Niromanesh S Shirazi M Eftekhariyazdi M Mortazavi F Comparison of umbilical artery Doppler and non-stress test in assessment of fetal well-being in gestational diabetes mellitus: A prospective cohort study Electron Physician 2017 9 12 6087 6093 10.19082/6087 29560164 PMC5843438

[122] Malhotra N Chanana C Kumar S Roy K Sharma J B Comparison of perinatal outcome of growth-restricted fetuses with normal and abnormal umbilical artery Doppler waveforms Indian J Med Sci 2006 60 08 311 317 10.4103/0019-5359.26607 16864917

[123] Lees C Perinatal and 2 year neurodevelopmental outcome in late preterm fetal compromise: the TRUFFLE 2 Randomised Trial [Study protocol] [Internet] 2020[cited 2021 Jan 12]. Available from:https://www.fundingawards.nihr.ac.uk/award/NIHR127976 10.1136/bmjopen-2021-055543 PMC9014041 35428631

[124] D'Antonio F Patel D Chandrasekharan N Thilaganathan B Bhide A Role of cerebroplacental ratio for fetal assessment in prolonged pregnancy Ultrasound Obstet Gynecol 2013 42 02 196 200 10.1002/uog.12357 23239502

